# Analysis of the expression pattern of the schizophrenia-risk and intellectual disability gene TCF4 in the developing and adult brain suggests a role in development and plasticity of cortical and hippocampal neurons

**DOI:** 10.1186/s13229-018-0200-1

**Published:** 2018-03-22

**Authors:** Matthias Jung, Benjamin M. Häberle, Tristan Tschaikowsky, Marie-Theres Wittmann, Elli-Anna Balta, Vivien-Charlott Stadler, Christiane Zweier, Arnd Dörfler, Christian Johannes Gloeckner, D. Chichung Lie

**Affiliations:** 1 Institute of Biochemistry, Emil Fischer Center, Friedrich-Alexander-Universität Erlangen-Nürnberg, 91054 Erlangen, Germany; 2 Institute of Human Genetics, Friedrich-Alexander-Universität Erlangen-Nürnberg, Erlangen, Germany; 3 Department of Neuroradiology, University Clinic Erlangen, Friedrich-Alexander-Universität Erlangen-Nürnberg, 91054 Erlangen, Germany; 4 German Center for Neurodegenerative Diseases, 72076 Tübingen, Germany; 5 Institute for Ophthalmic Research, Center for Ophthalmology, University of Tübingen, 72076 Tübingen, Germany

**Keywords:** Pitt-Hopkins syndrome, Schizophrenia, TCF4, Neurodevelopment

## Abstract

**Background:**

Haploinsufficiency of the class I bHLH transcription factor TCF4 causes Pitt-Hopkins syndrome (PTHS), a severe neurodevelopmental disorder, while common variants in the *TCF4* gene have been identified as susceptibility factors for schizophrenia. It remains largely unknown, which brain regions are dependent on TCF4 for their development and function.

**Methods:**

We systematically analyzed the expression pattern of TCF4 in the developing and adult mouse brain. We used immunofluorescent staining to identify candidate regions whose development and function depend on TCF4. In addition, we determined TCF4 expression in the developing rhesus monkey brain and in the developing and adult human brain through analysis of transcriptomic datasets and compared the expression pattern between species. Finally, we morphometrically and histologically analyzed selected brain structures in *Tcf4*-haploinsufficient mice and compared our morphometric findings to neuroanatomical findings in PTHS patients.

**Results:**

TCF4 is broadly expressed in cortical and subcortical structures in the developing and adult mouse brain. The TCF4 expression pattern was highly similar between humans, rhesus monkeys, and mice. Moreover, *Tcf4* haploinsufficiency in mice replicated structural brain anomalies observed in PTHS patients.

**Conclusion:**

Our data suggests that TCF4 is involved in the development and function of multiple brain regions and indicates that its regulation is evolutionary conserved. Moreover, our data validate *Tcf4*-haploinsufficient mice as a model to study the neurodevelopmental basis of PTHS.

## Background

The transcription factor 4 (TCF4, Gene ID: 6925) and its paralogues TCF3 and TCF12 form the subgroup of class I basic Helix-Loop-Helix (bHLH) transcription factors (TFs). Class I bHLH TFs are ubiquitously expressed, and their transcriptional output is dependent on their interaction partners. They are obligate partners for proneural class II bHLH TFs, which are unable to exert transcriptional function without dimerization to Class I bHLH. Class I bHLH TF are inhibited by interaction Class V bHLH factors, which do not possess a DNA-binding domain and sequester class I factors [1].

TCF4 has gained major attention following the discoveries that heterozygote loss-of-function mutations in *TCF4* cause Pitt-Hopkins syndrome (PTHS) [2, 3]—a neurodevelopmental disorder characterized by early onset developmental delay, moderate to severe intellectual disability, autistic behavior, intermittent breathing abnormalities, seizures, and distinctive facial features—and that single nucleotide polymorphisms (SNPs) in potentially regulatory regions of *TCF4* are associated with an increased risk of schizophrenia [4–6].

The extent of cognitive deficits in PTHS suggests that TCF4 dosage is critical for the development of various brain regions, but it remains largely unknown which neural populations develop in a TCF4-dependent fashion. Based on the rationale that TCF4 expression will predict regions that require TCF4 activity for their development, we performed a systematic analysis of the temporo-spatial expression pattern of TCF4 protein in mouse brain development. Moreover, we determined TCF4 expression in the adult mouse brain to identify neural systems, in which TCF4 may serve functions in maintenance and plasticity. Our analyses reveal that TCF4 is broadly expressed in cortical and subcortical structures in the development and adulthood with prominent expression in the cortex, hippocampus, and a subset of hypothalamic and amygdaloid nuclei. Comparison of the developmental and adult murine expression pattern with transcriptomic datasets from rhesus monkey and human brain revealed strong similarities in TCF4 expression between species. Moreover, we found that *Tcf4* haploinsuffiency in mice replicated structural brain anomalies that have been observed in individuals with PTHS. Thus, our data suggest that TCF4 regulation and function in brain development may be evolutionary conserved.

## Results

**Fig. 1:**
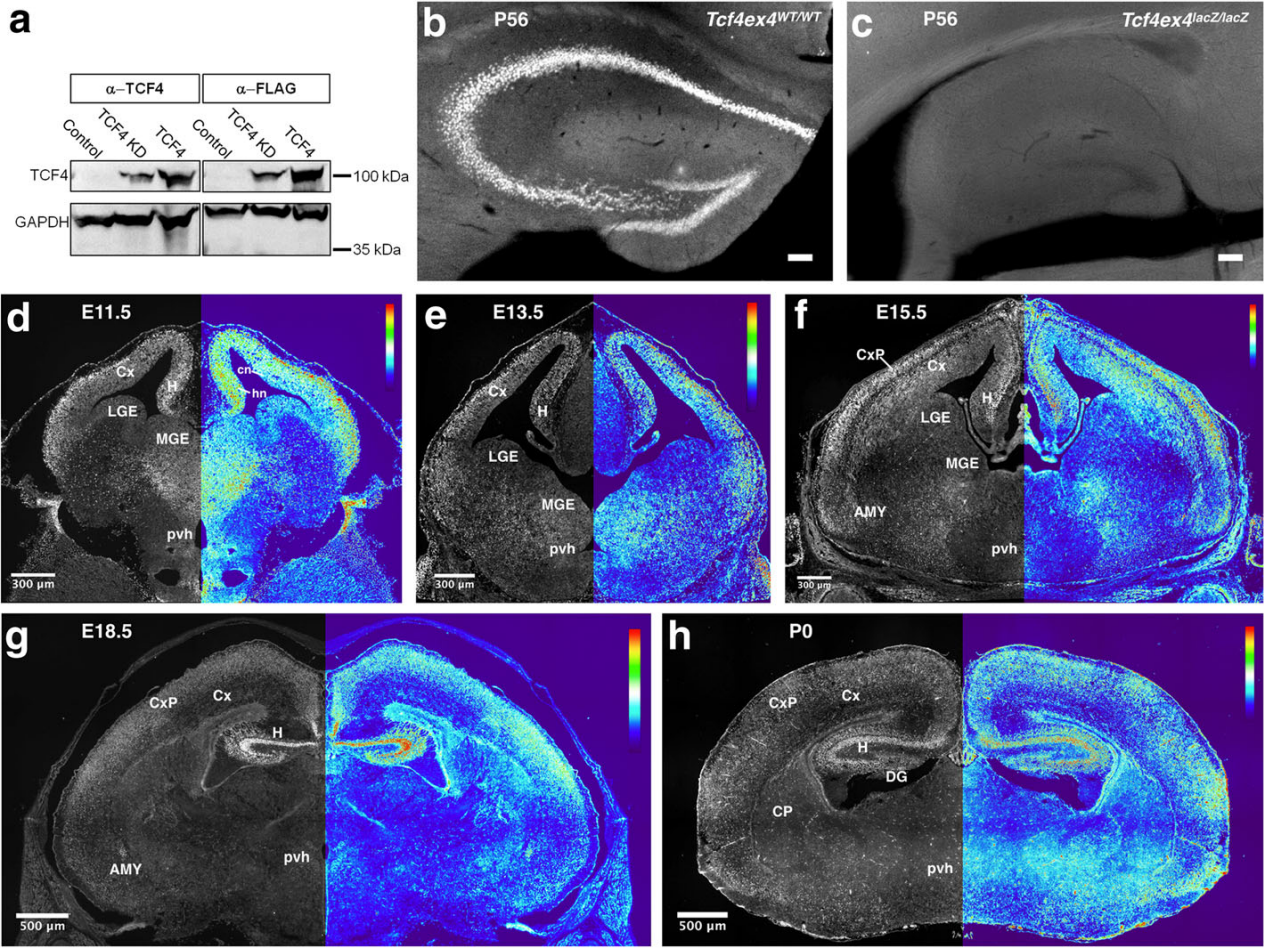
TCF4 expression at different developmental stages and validation of antibody specificity. **a** Western blot analysis of HEK 293T transfected with either CAG-GFP-IRES-GFP (Control), CAG-TCF4-IRES-GFP/ CAG-shTCF4-IRES-GFP (TCF4 KD), or CAG-TCF4-IRES-GFP (TCF4). Detection using the anti-TCF4 antibody revealed a clear band in the TCF4 transfected cells, which was reduced in size in the TCF4 KD (left panel). Detection using an anti-FLAG antibody resulted in bands equal in height compared to the TCF4 staining and also showed reduction upon TCF4 KD (right panel). GAPDH was used as the loading control. **b**, **c** Immunostaining using the anti-TCF4 antibody of brain tissue of TCF4 wildtype (WT) and constitutive TCF4-KO mice. **d**–**h** Left-hand side of each image, TCF4 immunostaining. Right-hand side, heatmap converted image. Relative immunoreactivity scale is shown in the upper right corner: red and blue represent high and low immunoreactivity, respectively. Abbreviations: AMY, amygdala; cn, cortical neuroepithelium; CP, caudoputamen; Cx, cortex; CxP, cortical plate; DG, dentate gyrus; H, hippocampal formation; hn, hippocampal neuroepithelium; LGE, lateral ganglionic eminence; MGE, medial ganglionic eminence; pvh, paraventricular hypothalamus

We first determined the specificity of the Tcf4-antibody intended for immunohistochemical analysis. To this end, HEK 293T cells were transfected with a C-terminally Flag-tagged TCF4-expression construct or with a control expression construct. The anti-TCF4-antibody as well as the anti-Flag antibody detected a single 100 kDa band in cell lysates from TCF4-transfected cells. The 100 kDa band was absent from cell lysates of control-transfected cells and was reduced in cells, in which a *TCF4*-specific shRNA construct was co-transfected with the *TCF4*-expression construct (Fig. 1a). Homozygous *Tcf4*-knockout (*Tcf4*^*lacZ/lacZ*^) is associated with high perinatal lethality, and from our breedings, we obtained only one *Tcf4*^*lacZ/lacZ*^ mouse that survived into adulthood. We used brain tissue from this mouse to further validate specificity of the TCF4-antibody. Staining with the TCF4 antibody yielded a robust signal in tissue from wildtype mice but no signal in homozygote *Tcf4*-knockout tissue (Fig. 1b, c). Collectively, these data demonstrate the specificity of the antibody for TCF4.

Next, we systematically analyzed the expression pattern of TCF4 protein in the developing and adult mouse brain. On embryonic day 11.5 (E11.5), TCF4 was broadly expressed in the germinal regions for cortical glutamatergic neurons and GABAergic neurons. Immunoreactive intensity varied between the distinct germinal regions. It was high in the cortical and hippocampal neuroepithelium and remained intense in the expanding neocortex and in the developing hippocampal formation (Fig. 1d–h). The ganglionic eminences are the main germinal zones for GABAergic neurons. The lateral ganglionic eminence (LGE) generates GABAergic interneurons and projection neurons of the olfactory bulb, amygdala, and striatum from E12 until birth [7, 8], while the medial ganglionic eminence (MGE) generates the majority of cortical interneurons between E12 and E16.5. MGE-derived interneuron precursors migrate tangentially to their final neocortical location [9]. Notably, the expression of TCF4 in the MGE correlated with the time of cortical interneuron production [10] (Fig. 1d–f). Moreover, TCF4 expression showed a lateral to medial gradient with moderate to high levels in the most medial part of the MGE and low to absent immunoreactivity in the LGE (Fig. 1d–f).

**Fig. 2:**
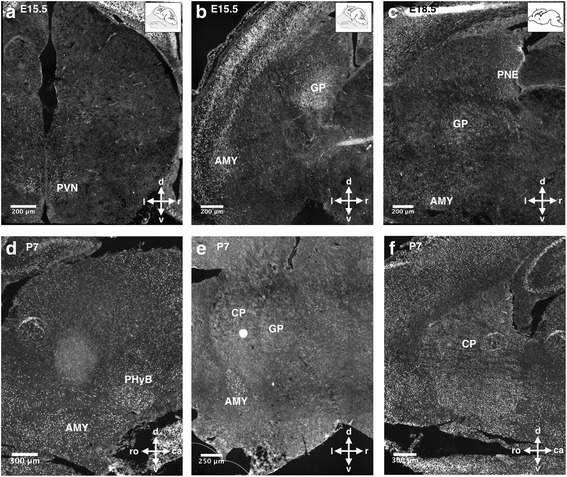
TCF4 expression in subcortical areas during neuronal development. TCF4 is expressed in developing hypothalamic nuclei (**a**), the globus pallidus (**b**, **c**, **e**), the amygdala (**b**, **c**, **d**, **e**), and the pallidal neuroepithelium (**c**). TCF4 immunoreactivity is also observed in the developing caudoputamen (**e**, **f**). Abbreviations: AMY, amygdala; PHyB, basal peduncular hypothalamus; CP, caudoputamen; GP, globus pallidus; MGE, medial ganglionic eminence; PVN, paraventricular hypothalamic nucleus; PNE, pallidal neuroepithelium

**Fig. 3:**
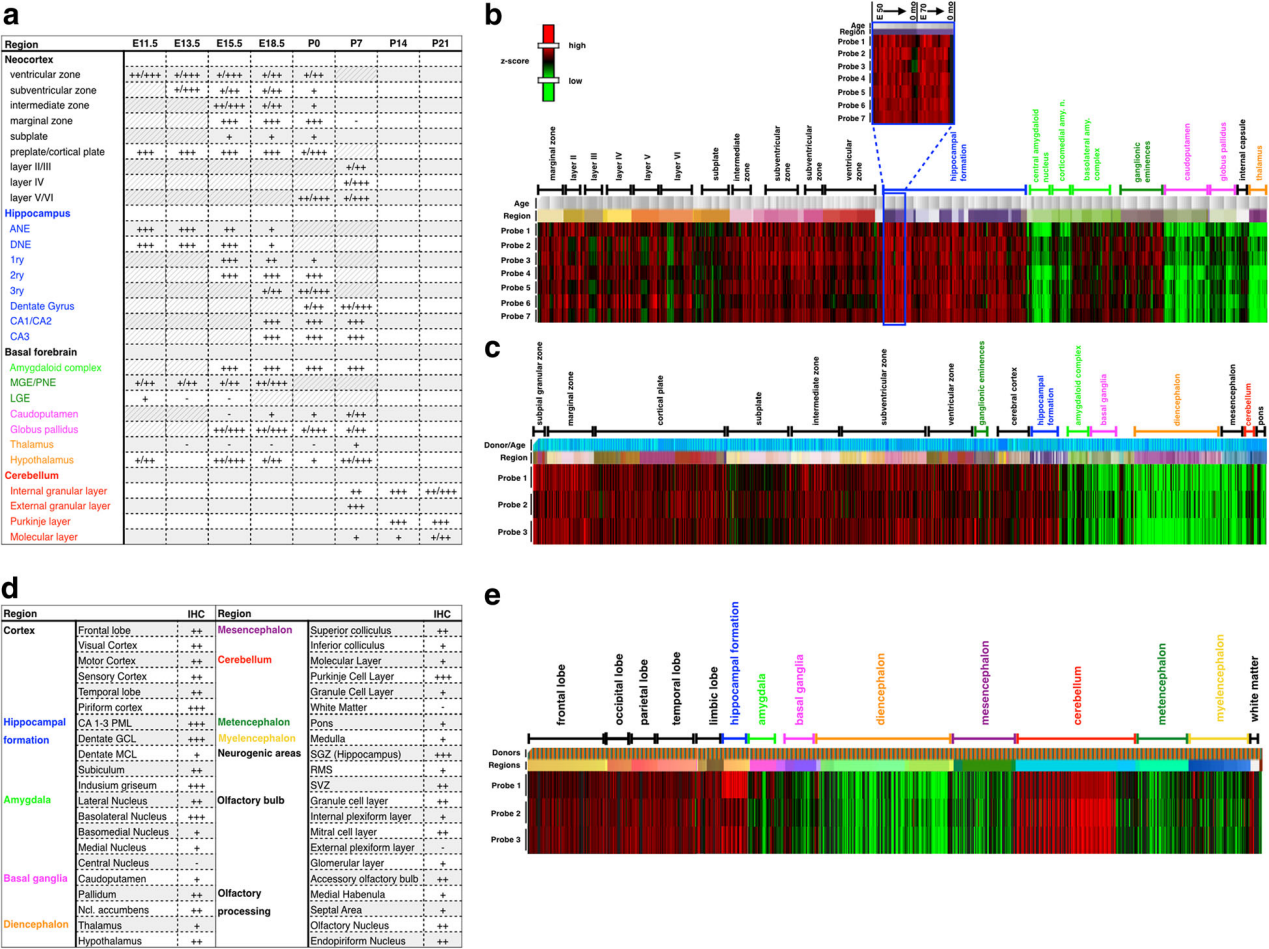
Comparison of developmental and adult TCF4 expression. **a** Tabular overview of TCF4 expression during neural development. +++, high. ++, moderate. +, low. -, not detected. Hatched cell: structure not defined at developmental stage. Empty cell: expression not determined. **b** TCF4 expression in selected brain areas during non-human primate neural development from E40–0 months. Microarray data of six probes is grouped by brain regions and arranged by ascending age per region (blow-up) (The NIH Blueprint Non-Human Primate (NHP) Atlas, http://www.blueprintnhpatlas.org). **c** TCF4 expression in selected brain areas during human neural development from post-conception week 15–21. Microarray data of three probes are grouped by brain regions and aligned by ascending age per region (BrainSpan: Atlas of the Developing Human Brain [Internet]. Funded by ARRA Awards 1RC2MH089921-01, 1RC2MH090047-01, and 1RC2MH089929-01. © 2011. Available from: http://www.brainspan.org/lcm/search?search_type=user_selections). **d** Tabular overview of TCF4 expression in the adult mouse brain (P56). **e** Brain region-specific TCF4 expression in the adult human brain [23]

Regionally distinct TCF4 expression levels were observed in subcortical areas. Developing hypothalamic nuclei showed moderate expression of TCF4 starting from E11.5 onwards (Fig. 1d–h); at E15.5 and postnatal day 7 (P7), TCF4 showed prominent expression in a subset of cells in the paraventricular hypothalamic nucleus and the basal peduncular hypothalamus, respectively (Fig. 2a, d). From E15.5, when the amygdaloid complex can be clearly distinguished, we found continuously high expression of TCF4 in this region (Fig. 2b–e). In the globus pallidus (GP), TCF4 expression was high at E15.5 but decreased at subsequent stages (Fig. 2b, c, e). Scattered cells with low to intermediate TCF4 expression were observed in the caudoputamen (CP) at postnatal stages (Fig. 1h; Fig. 2e, f). A summary of the developmental expression pattern and levels of TCF4 is provided in Fig. 3a.

**Fig. 4:**
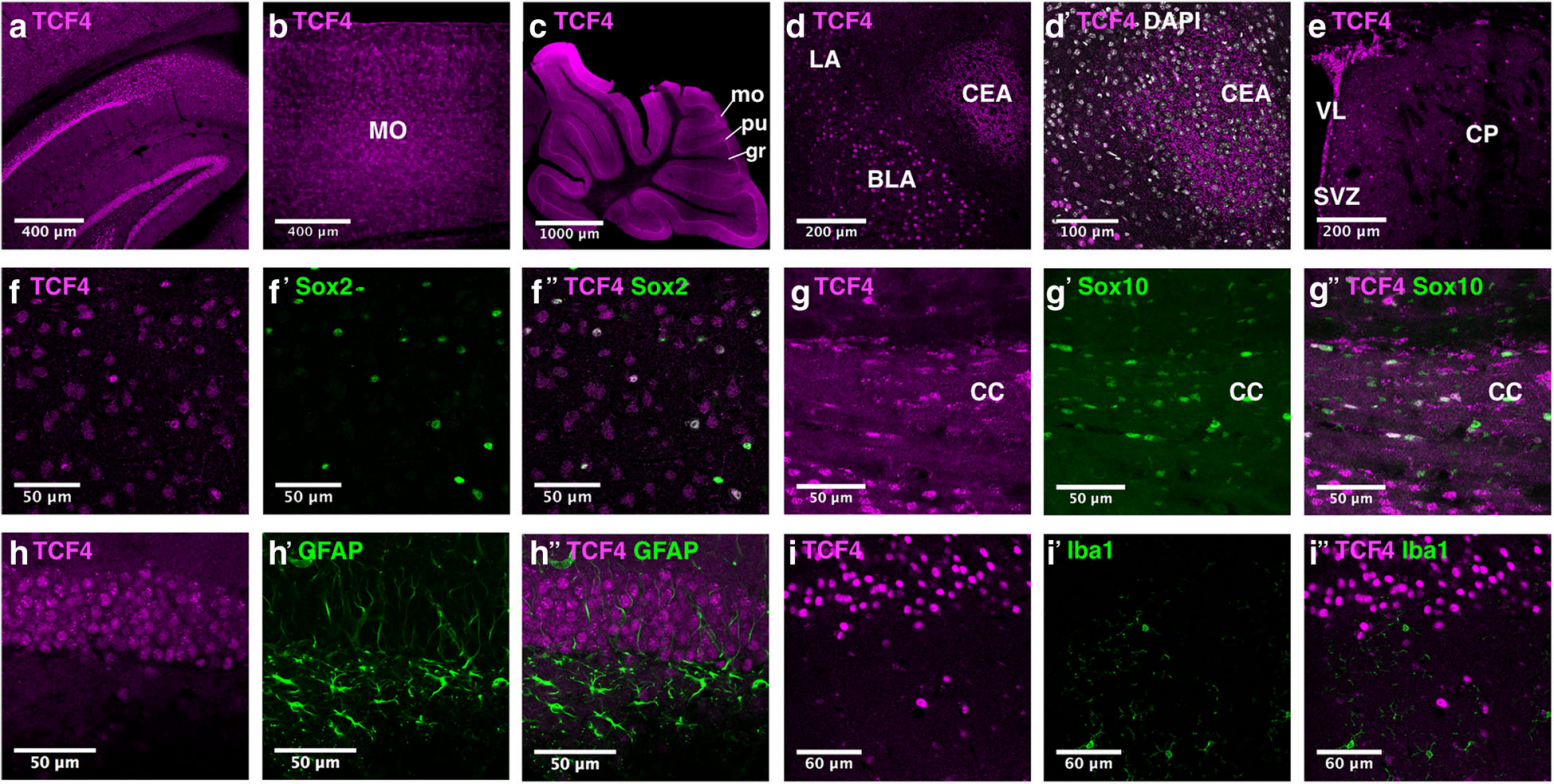
TCF4 expression in regions and non-neuronal cell types of the adult brain. In the adult brain, TCF4 is expressed in the hippocampus (**A**), the cortex (**A**, **B**), the cerebellum (**C**), the amygdaloid complex (**D**–**D’**) and the caudoputamen (**E**). TCF4 immunoreactivity is observed in astrocytes (**F**–**F”**, **H**–**H”**) and oligodendrocytes (**G**–**G”**) but not in microglial cells (**I**–**I”**). Abbreviations: BLA basolateral amydala nucleus; CC, corpus callosum; CEA, central amygdala nucleus; CP, caudoputamen; gr, granule cell layer; LA, lateral amygdala. MO, somatomotor areas; mo, molecular cell layer; pu, purkinje cell layer nucleus; SVZ, subventricular zone; VZ, ventricular zone

In young adult mice (P56), the highest TCF4 immunoreactivity was found in the hippocampal formation, the cortex, the purkinje cell layer of the cerebellum, and the amygdala (Fig. 4A–D’). Of the amygdala subnuclei, cells of the lateral (LA) and basolateral (BLA) nucleus highly express TCF4. The central nucleus (CEA) also shows high TCF4 immunoreactivity; in contrast to the LA and BLA nuclei, TCF4 signal in the CEA was not nuclear but was located in fibers, whose distribution strongly resembled the distribution pattern of calcitonin gene-related peptide immunoreactive projections that arise from posterior thalamic nuclei [11]. Low to intermediate TCF4 immunoreactivity was found in structures of the diencephalon, the midbrain, and in scattered cells of the caudoputamen (Fig. 4E). The expression pattern of TCF4 in the adult mouse brain is summarized in Fig. 3d.

Next, we analyzed the expression of TCF4 in glial cells. TCF4 was expressed in the majority of astroglia and some Sox10-positive oligodendroglia (Fig. 4F–H”). Expression levels in both glial populations varied from low to high. *Iba1*-positive microglial cells did not show TCF4 immunoreactivity (Fig. 4F–I”).

**Fig. 5:**
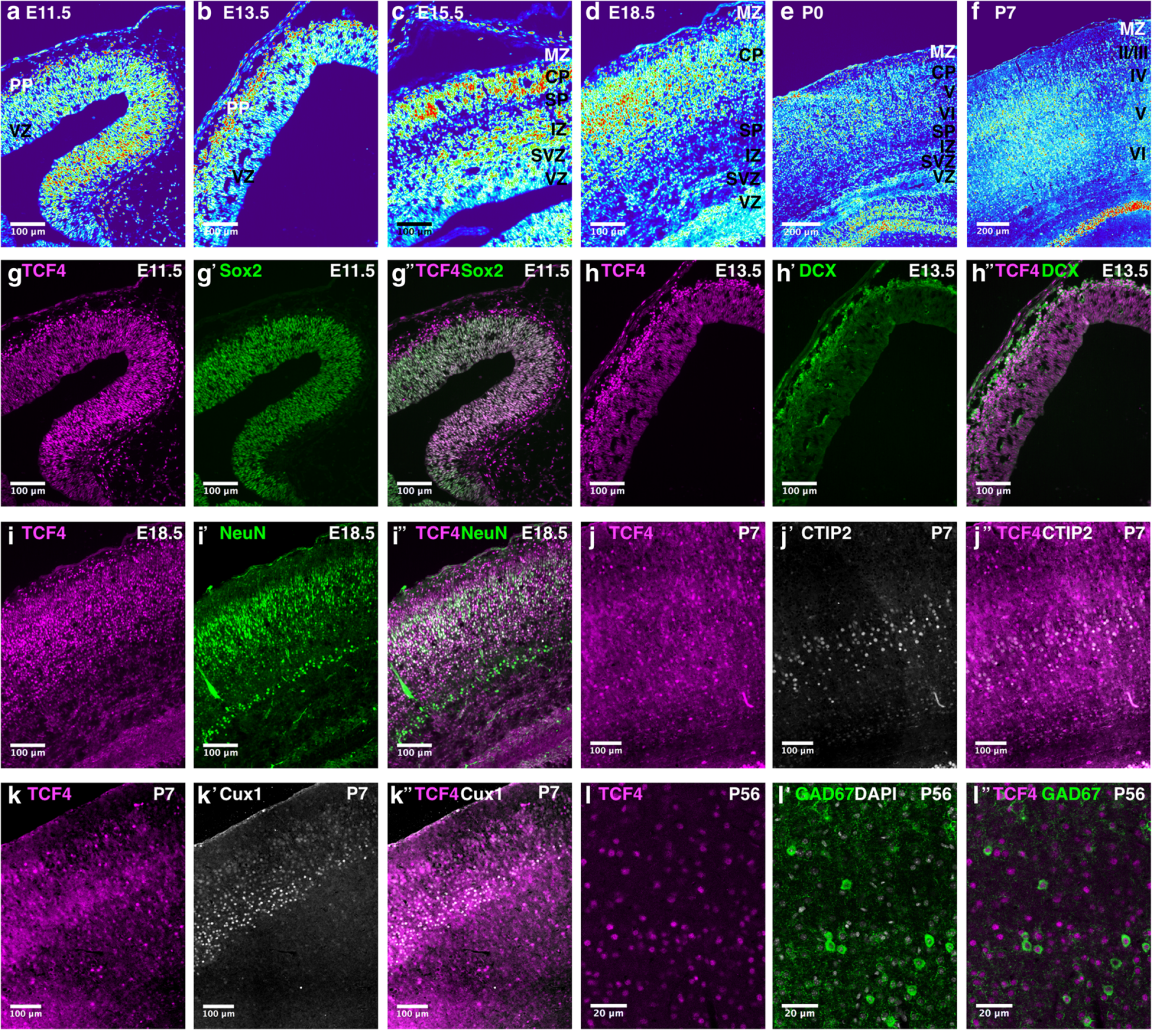
TCF4 expression during corticogenesis. (**A**–**F**) Heatmap converted TCF4 immunoreactivity at different stages of cortical development. TCF4 is expressed in all layers of the neocortex with particular high intensity in the expanding cortical plate during development (**G**–**I”**). TCF4 is expressed in Sox2-positive ventricular zone cells (**G**–**G”**), DCX-positive immature neurons (**H**–**H”**), and NeuN-positive neurons (**I**–**I”**). TCF4 co-stainings with layer V/VI neuron marker CTIP2 (**J**–**J”**) or layer II/III/IV neuron marker Cux1 (K-K”) at P7. TCF4 is expressed in layers II-VI but only individual TCF4-positive cells co-localize with the layer markers. TCF4 is expressed in GAD67-positive interneurons (**L**–**L”**). Abbreviations: CP, cortical plate; IZ, intermediate zone; MZ, marginal zone; PP, preplate; SP, subplate; SVZ, subventricular zone; VZ, ventricular zone

Given the prominent expression of TCF4 in the cortex and hippocampus, we analyzed these areas in more detail. During cortical development, radial glial cells (RGCs) in the ventricular zone (VZ) divide to self-renew and to generate either immature neurons or intermediate progenitor cells (IPCs) [12, 13]. IPCs accumulate in the subventricular zone (SVZ) where they give rise to postmitotic neurons via asymmetric cell division [13]. Neurons migrate radially into the expanding cortical plate (CP), forming the distinct cortical layers in an inside-out fashion with later-born excitatory neurons migrating to more superficial layers of the CP passing over earlier born neurons [14, 15]. At E11.5, TCF4 expression is high in the ventricular zone (VZ), which coincides with the generation of preplate (PP) neurons from neural progenitors in this region (Fig. 5A). Co-staining with the precursor marker Sox2 confirmed that TCF4 was indeed highly expressed by VZ precursors at this time point (Fig. 5G–G”). At later stages (E13.5 and thereafter), TCF4 immunoreactivity was low to moderate in the germinal zones, i.e., the VZ and SVZ, but was high in the PP and the expanding cortical plate (Fig. 5B–D). At E13.5, the highest TCF4 expression levels co-localized with DCX, a marker for immature migrating neurons (Fig. 5H–H”); at E18.5, high TCF4 expression levels frequently co-localized with NeuN, a marker for post-migratory neurons (Fig. 5I–I”). Cells with high TCF4 expression were also observed in the postnatal and adult cortex (Fig. 5E, F, J–L”). These TCF4-expressing cells also expressed the layer V/VI neuronal marker CTIP2 or the layer II-IV neuronal marker Cux1, demonstrating that subsets of cortical neurons expressed high levels of TCF4 (Fig. 5J–K”). In addition, high TCF4 expression was observed in a subset of GAD67-positive cortical interneurons (Fig. 5L–L”).

**Fig. 6:**
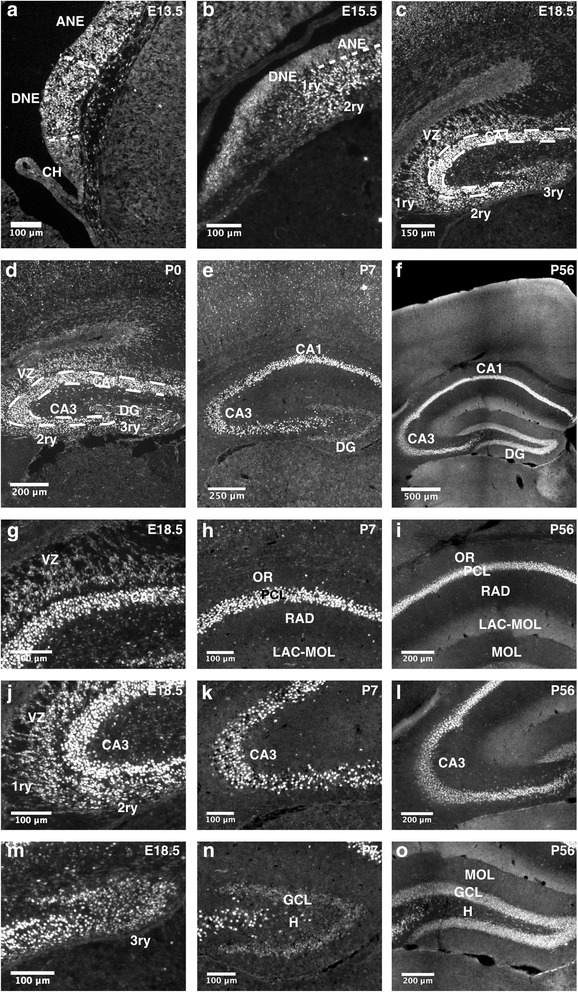
TCF4 expression during hippocampal development. **a**–**f** Overview images of the hippocampal formation at different developmental stages. TCF4 is expressed throughout pre- and postnatal development of the hippocampus. **g**–**i** TCF4 expression in the CA1 region of the hippocampus. At E18.5, TCF4 is expressed in radially migrating pyramidal progenitor cells and in the forming pyramidal cell layer. Postnatally, TCF4 is expressed in the pyramidal cell layer and in scattered cells in the stratum oriens and radiatum. **j**–**l** At E18.5, TCF4 is expressed in dentate progenitor cells that migrate to the subpial zone (primary and secondary matrix) and in the forming pyramidal cell layer of the CA3 region. At postnatal stages P7 and P56, TCF4 is expressed in the pyramidal cell layer and in scattered cells of the strata oriens and radiatum. **m**–**o** At E18.5, TCF4 is expressed in dentate progenitor cells that accumulate in the hippocampal fissure (tertiary matrix). Postnatally, TCF4 is expressed in the granular cell layer of the dentate gyrus and in individual cells of the hilus. Abbreviations: 1ry, primary matrix; 2ry, secondary matrix; 3ry, tertiary matrix; ANE, ammonic neuroepithelium; CH, cortical hem; DG, dentate gyrus; DNE, dentate neuroepithelium; GCL, granular cell layer; H, hilus; LAC-MOL, stratum lacunosum-moleculare; MOL, molecular layer; OR, stratum oriens; PCL, pyramidal cell layer; RAD, stratum radiatum; SVZ, subventricular zone

**Fig. 7:**
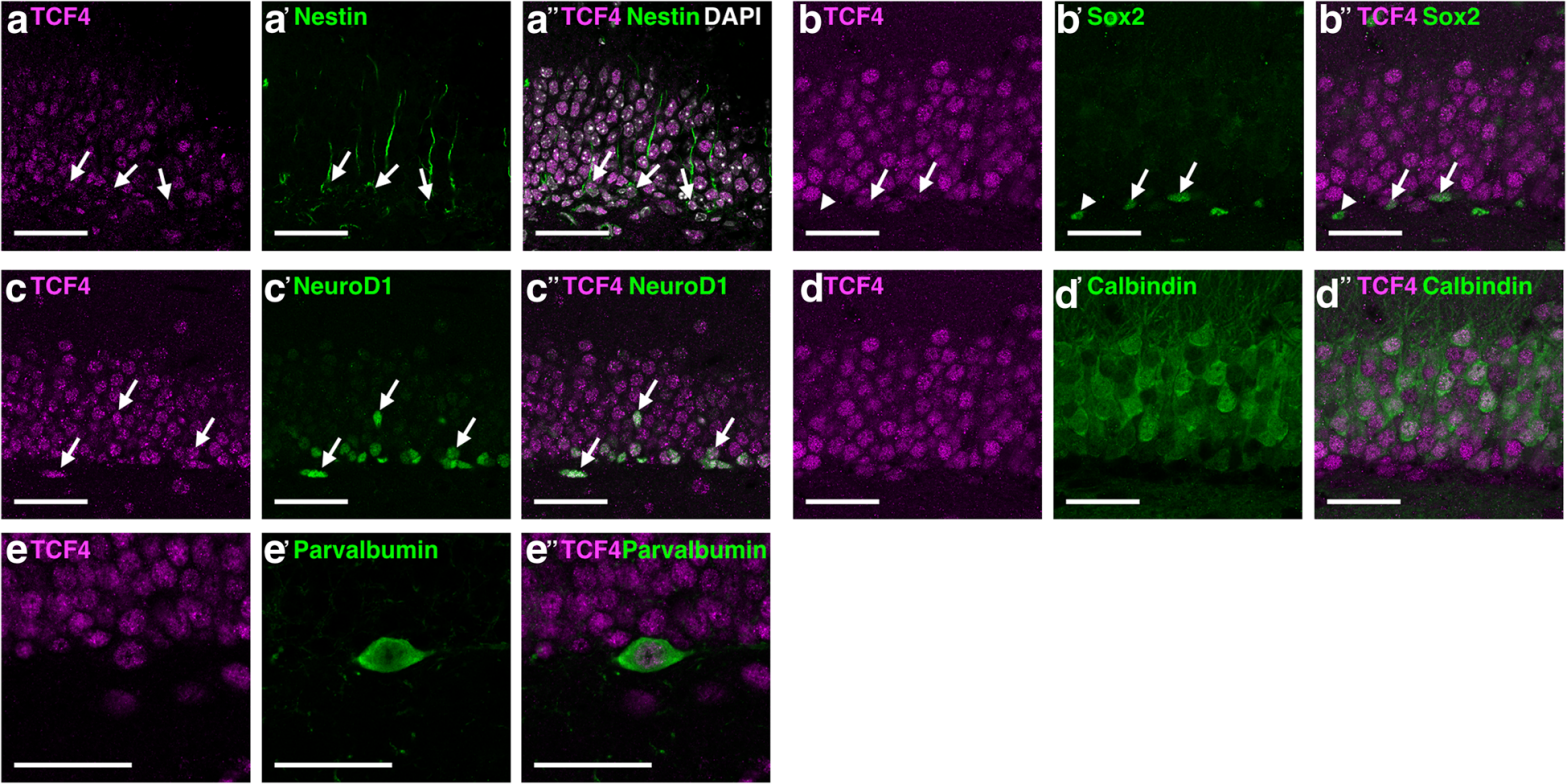
TCF4 expression in different cell types in the adult dentate gyrus. **A**–**A”** TCF4 is expressed in Nestin-positive radial glia-like cells. **B**–**B”** TCF4 is partially expressed in Sox2-positive cells. **C**–**C”** TCF4 is expressed in NeuroD-positive immature neurons, **D**–**D”** TCF4 is expressed in Calbindin-positive mature neurons, and **E**–**E”** in Parvalbumin-positive interneurons. Arrowheads mark TCF4 negative/cell type marker positive cells. Scale bars are 40 μm

The hippocampal neuroepithelium is the germinal zone for hippocampal neurons and is subdivided into the Ammon’s horn neuroepithelium (ANE) and the dentate gyrus neuroepithelium (DNE). CA1-CA3 pyramidal neuron precursors are generated in the ANE and migrate radially from the VZ to the prospective CA1-CA3 subfields where they undergo terminal differentiation and maturation. The DNE, which is also called primary matrix, generates dentate granule neuron precursors (secondary matrix) that migrate along a radial glial scaffold to the subpial zone of the hippocampus [16–19]. Reaching the hippocampal fissure, precursors accumulate and form the tertiary matrix [16]. During the first postnatal week, the secondary matrix disappears, while the tertiary matrix remains the proliferative zone generating the inner shell of the granule cell layer [16, 19]. From P14 onwards, the tertiary matrix resolves and the neurogenic niche becomes successively confined to the subgranular zone (SGZ) of the dentate gyrus (DG) [16, 20]. TCF4 is highly expressed in pyramidal and dentate granule precursors and neurons during the course of hippocampal development (Fig. 6a–f). In contrast to the adult cortex, where high TCF4 expression is observed only in a subset of neurons, TCF4 expression in the adult hippocampus remained prominent throughout the CA subfields and the dentate gyrus (Fig. 6g–o). In the dentate gyrus, TCF4 was not only strongly expressed in Calbindin-positive mature dentate granule neurons but also in Parvalbumin-positive interneurons (Fig. 7D–E”). The dentate gyrus is one of two regions of the adult mammalian brain, where stem cells generate neurons throughout life [21]. Nestin-positive radial glia-like stem cells in the subgranular zone displayed moderate TCF4 immunoreactivity (Fig. 7A–A”). Sox2-positive precursors and astroglia in the SGZ showed variable levels of TCF4 expression, whereas NeuroD1-positive immature dentate granule neurons showed strong TCF4 immunoreactivity (Fig. 7B–C”). Collectively, the expression data of TCF4 strongly suggests that TCF4 is intimately linked to developmental and adult hippocampal neurogenesis. The persistence of high TCF4 expression in mature neurons of all hippocampal subfields raises the intriguing possibility that TCF4 is also involved in hippocampal plasticity.

Next, we sought to evaluate whether the TCF4 expression pattern is conserved from mice to humans. The NIH Blueprint Non-Human Primate (NHP) Atlas (2009) (http://www.blueprintnhpatlas.org) provides mRNA expression data of the developing rhesus monkey brain from E40–0 months; the BrainSpan Atlas documents mRNA expression data of human neural development for post conception weeks 15–21 [22]. *TCF4* mRNA expression data in the adult human brain was extracted from the Allen Human Brain Atlas (© 2010 Allen Institute for Brain Science. Allen Human Brain Atlas. Available from: human.brain-map.org, [23]). The neurodevelopmental expression pattern of *TCF4* was highly similar between mice, rhesus monkeys, and humans: regions with high expression during murine neural development such as the hippocampus, the cortex, the ganglionic eminences, and some nuclei of the amygdaloid complex, consistently show high *TCF4* expression in the developing brain of rhesus monkey and humans, whereas low *TCF4* expressing regions such as the caudoputamen also show low expression during rhesus monkey and human brain development (Fig. 3a-c).

Striking similarities in the TCF4 expression pattern were also found during adulthood. Regions with high expression in adult mice, i.e., hippocampus, cerebellum, cortex, and nuclei of the amygdaloid complex, were also the highest *TCF4*-expressing regions in the adult human brain, while, for example, the diencephalon, metencephalon, myelencephalon, and parts of the basal ganglia showed low *TCF4* expression levels in humans and mice (Fig. 3d, e). These data indicate that the expression pattern of TCF4 is conserved from mouse to humans.

**Fig. 8:**
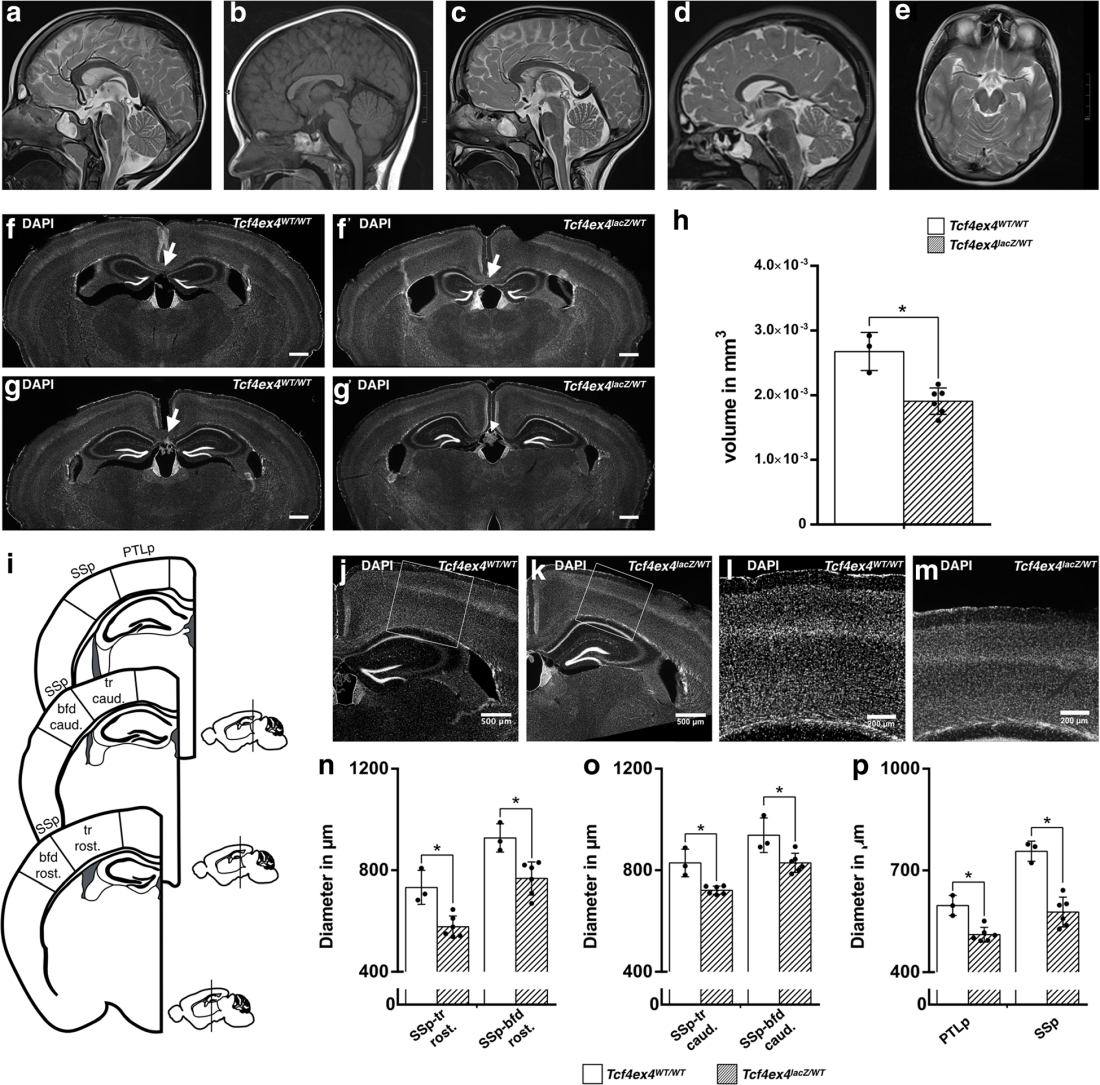
PTHS-associated brain anomalies in TCF4-haploinsufficient mice. Hypoplastic corpus callosum in *Tcf4*-haploinsufficient PTHS patients at the age of 3 years (**B**) and 7 years (**D**). **A**, **C** Age-matched references. **E** Small hippocampus in PTHS patient at the age of 7 years. *Tcf4*-haploinsufficient mice (**F’**, **G’**) show agenesis of the corpus callosum and reduced hippocampal volume (**H**) compared to WT mice (**F**, **G**). Cortical diameter was determined along the rostro-caudal axis (**I**). Representative images of the cortical thickness of *Tcf4ex4*^*WT/WT*^ mice (**J**, **L**) and *Tcf4ex4*^*lacZ/WT*^ mice (**K**, **M**). Cortical diameter of *Tcf4ex4*^*lacZ/WT*^ mice was significantly decreased compared to *Tcf4ex4*^*WT/WT*^ mice in all analyzed cortical regions. The results are displayed as mean in microns ± SD. **N**–**P** Scale bars 500 μm. The DG area was measured in six representative slices spanning a distance of 240 μm. Abbreviations: PTLp, posterior parietal association area; SSp-bfd, primary somatosensory area-barrel field; SSp-tr, primary somatosensory area-trunk; Rost, rostral; Caud, caudal

In humans, *TCF4* haploinsufficiency causes Pitt-Hopkins syndrome, a severe neurodevelopmental disorder, associated with psychomotor delay, intellectual disability, and autistic behavior. MRI-based findings of structural anomalies in PTHS are variable, ranging from normal cerebral MRIs (30–50% of cases) to enlarged ventricles, cerebellar atrophy, hippocampal hypoplasia, and hypoplasia of the corpus callosum [24–26]. We analyzed MRIs of two individuals with genetically confirmed PTHS. Consistent with previous studies reporting hypoplasia of the corpus callosum as a frequent structural anomaly in PTHS (24–45% of cases), we found general thinning of the corpus callosum and aplasia of the splenium of the corpus callosum in both individuals (Fig. 8A–D). One of the individuals also displayed a small hippocampal formation (Fig. 8E).

We next investigated whether *Tcf4* haploinsufficiency in mice reproduced structural anomalies of PTHS. To determine the influence of *Tcf4*-haploinsufficiency on cortical architecture, we analyzed the diameter of six different cortical regions along the rostral to caudal axis. Cortical thickness was significantly reduced in all analyzed areas in *Tcf4*-haploinsufficient (*Tcf4ex4*^*lacZ/WT*^) mice compared to their wildtype littermates (WT) (Fig. 8I–P). Moreover, the dentate gyrus volume was significantly decreased in *Tcf4ex4*^*lacZ/WT*^ mice (Fig. 8F–H). In addition, *Tcf4ex4*^*lacZ/WT*^ mice displayed agenesis of the splenium of the corpus callosum (Fig. 8F–G’). Thus, *Tcf4* haploinsufficiency in mice results in anatomical deficits that resemble anatomical anomalies observed in PTHS.

**Fig. 9:**
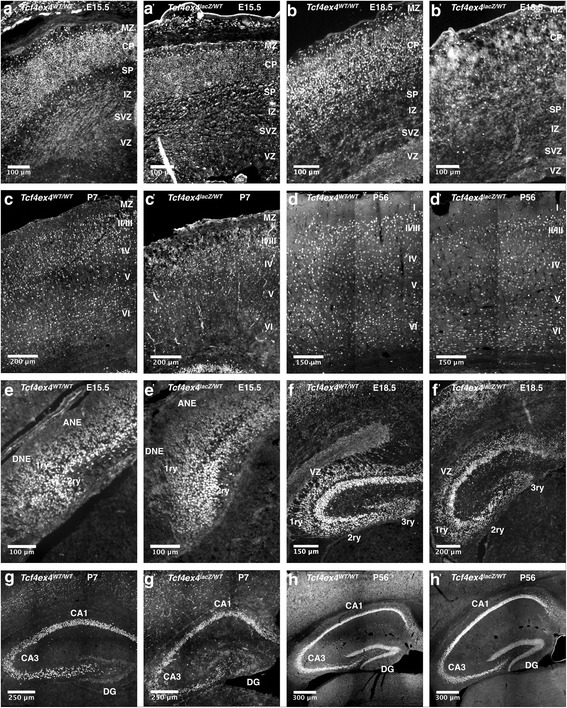
Comparison of TCF4 protein expression at different stages of cortical and hippocampal development between *Tcf4ex4*^*WT/WT*^ and *Tcf4ex4*^*lacZ/WT*^ mice. **A**–**D’** Immunostaining using the anti-TCF4 antibody on brain tissue of *Tcf4ex4*^*WT/WT*^ and *Tcf4ex*^*lacZ/WT*^ animals during cortical development and in the adult. **E**–**H’** Immunostaining using the anti-TCF4 antibody on brain tissue of *Tcf4ex4*^*WT/WT*^ and *Tcf4ex4*^*lacZ/WT*^ animals during hippocampus formation and in the adult. Abbreviations: ANE, ammonic neuroepithelium; CP, cortical plate; DG, dentate gyrus; DNE, dentate neuroepithelium; IZ, intermediate zone; MZ, marginal zone; PP, preplate; SP, subplate; SVZ, subventricular zone; VZ, ventricular zone; 1ry, primary matrix; 2ry, secondary matrix; 3ry, tertiary matrix

Finally, we qualitatively compared TCF4-expression in the cortex and hippocampus at E15.5, E18.5, P7, and P56 between WT and *Tcf4*-haploinsufficient mice (Fig. 9) The cortex of *Tcf4ex4*^*lacZ/WT*^ animals appeared markedly thinner at all time points (Fig. 9A–D’). The density of TCF4-expressing cells in the cortex of *Tcf4ex4*^*lacZ/WT*^ mice seemed to be reduced, and TCF4-expressing cells showed a less regular distribution. Similarly, the arrangement of TCF4-expressing cells in the developing hippocampus (E15.5–P7) of *Tcf4ex4*^*lacZ/WT*^ mice was less organized (Fig. 9E–H’). Moreover, TCF4-expressing cells in the CA3 subfield of adult *Tcf4*-haploinsufficient mice appeared to be less densely packed (Fig. 9H–H’).

## Discussion

Here, we used immunohistochemical analysis to systematically investigate the expression of the intellectual disability and schizophrenia-associated *Tcf4* gene in the developing and adult murine CNS. Consistent with recent experimental evidence linking TCF4 to precursor proliferation, neuronal positioning and development of excitability in the developing cortex [27–29], and the suggestion that TCF4 regulates neurite branching and synapse formation in prefrontal cortical neurons [30], we found TCF4 to be expressed from the early precursor stage up to the post-migratory neuronal stage in the developing cortex. We also demonstrate that TCF4 is expressed in the major germinal zones for the principal neurons of the hippocampus, for cortical interneurons, as well as in developing neurons of, for example, the cerebellum and some nuclei of the amygdaloid complex. The broad expression of TCF4 strongly suggests that TCF4 regulates early and late development of cortical and subcortical structures.

As a class I bHLH TF, the transcriptional output of TCF4 is dependent on interaction with other bHLH TFs. Through such interactions, TCF4 may act as a network hub coordinating different transcriptional modules and developmental processes. The interaction partners of TCF4 in the developing CNS have not been defined. The bHLH TFs Ascl1, Neurogenin 1 and 2, NeuroD1, and NeuroD6, have a restricted developmental expression pattern and fulfill defined stage-specific functions during hippocampal and cortical development [31–33]. It is tempting to speculate that TCF4 fulfills at least part of its functions in CNS development through interaction with these proneural class II bHLH TFs.

Recent work indicated that TCF4 activity is modulated by post-translational mechanisms such as neuronal activity-dependent phosphorylation [34]. Interestingly, we detected TCF4 immunoreactivity in fibers of the CEA, raising the possibility that TCF4 activity may be modulated by nuclear-cytoplasmatic translocation.

PTHS is associated with deficits in various functional domains including cognition, social interaction, speech, motor skills, and vegetative function, and there is some debate to which extent the PTHS phenotype may be due to post-developmental functions of TCF4 in mature neurons [35]. Indeed, it has been shown that TCF4 directly regulates plasticity of CA1 pyramidal neurons potentially through the control of activity-dependent DNA methylation [35]. Strikingly, we found that TCF4 is highly expressed not only in the hippocampus but also in a number of cortical and subcortical structures during adulthood, raising the possibility that TCF4 function in mature neurons is critical for plasticity in many neural circuits.

*TCF4* haploinsufficiency causes PTHS, while SNPs in regulatory regions, which may cause increased TCF4 expression [36], are associated with an elevated risk for schizophrenia. Moreover, behavioral deficits have been reported not only for *Tcf4*-haploinsufficient mice but also for transgenic mice with mild TCF4 over-expression [37, 38]. These observations have led to the suggestion that TCF4 levels have to be kept in a tight range to allow for neuronal development and function. Intriguingly, we observed cell type- and region-dependent differences in TCF4 expression, raising the questions whether distinct cell populations may require different TCF4 dosages and whether there is cell type-/region-specific vulnerability to pathologically altered TCF4 expression levels.

## Conclusion

A recent study reported similarities in the expression of *TCF4* mRNA in the developing prefrontal cortex of rats and humans [39]. Here, we show that the expression pattern of *TCF4* between mice and humans is highly similar across the developing and adult brain and provide evidence that *Tcf4* haploinsufficiency in mice reproduces structural anomalies that are observed in PTHS. These observations strongly suggest that the regulation and function of TCF4 is conserved between species and further validate *Tcf4*-haploinsufficient mice as a preclinical model for PTHS.

Our preliminary analysis indicated an irregular distribution of TCF4-expressing cells in the cortex and hippocampus of *Tcf4*-haploinsufficient mice strengthening the notion that TCF4 plays a role in migration and positioning of neurons [40]. A more detailed analysis of *Tcf4*-haploinsufficient mice will allow to decipher the pathophysiological mechanisms underlying PTHS on the molecular, cellular, and neural circuit level and to explore experimental strategies to ameliorate cognitive and behavioral deficits in this disorder [35, 41, 42].

## Methods

### Animal husbandry

All animal experiments were carried out in accordance with the European Communities Council Directive (86/609/EEC) and approved by the government of Middle-Franconia, Germany. C57Bl/6NRj mice were obtained from Janvier Labs (Le Genest-Saint-Isle, France) and group-housed under a 12 h light/dark cycle with ad libitum access to food and water. Tcf4ex4WT/lacZ mice were obtained from the Wellcome Trust Sanger Institute (Alleles produced for the EUCOMM and EUCOMMTools projects by the Wellcome Trust Sanger Institute; MGI ID: 4432303). For generation of the knockout allele, the L1L2_gt1 cassette was inserted at position 69461371 of Chromosome 18 upstream of the in silico-determined critical exon four. The L1L2_gt1 cassette harbors an FRT flanked lacZ/neomycin sequence followed by a loxP site resulting in a “knockout first” allele. An additional loxP site is inserted downstream of the targeted exon four at position 69462143 [39]. For embryonic studies, mice were bred in the afternoon and vaginal post-coitum protein plug check (“Plug check”) was performed the next morning. This time point was defined as E0.5.

### Tissue preparation and dissection

Timed pregnant mice were killed by cervical dislocation. E11.5 and E13.5 embryos were fixed overnight in 4% PFA. For the E15.5 time point, heads were fixed overnight in 4% PFA. For the E18.5 and P0 time points, brains were dissected and fixed overnight in 4% PFA. Tails were used for genotyping. After fixation tissue was washed repeatedly with 1× PBS and transferred to 30% sucrose in PBS overnight for dehydration. Embryonic tissues were embedded in freezing media (Jung, Nussloch) and stored at − 80 °C. Adult mice were killed using CO_2_ and transcardially perfused with PBS for 5 min (20 ml/min) followed by fixation with 4% paraformaldehyde (PFA) in PBS, pH 7.4, for 5 min. The brains were post-fixed overnight in 4% PFA at 4 °C followed by dehydration at 4 °C in 30% sucrose in TBS.

### Histology

Embryonic tissue was cut in 10 μm thin sections with a cryotom (Leica Microsystems, Wetzlar). Sections were transferred on laminated object slides and dried for 2 h at room temperature and stored at − 80 °C until further use. Sections were washed three times for 5 min with 1xPBS, treated with 50 mM citrate buffer at 70 °C for 3 min for antigen retrieval. Tissue was permeabilized for 10 min in 0.1% Triton-X/PBS and blocked with blocking solution (10% FCS, 1% BSA in PBS) at room temperature for 2 h in a wet chamber. Sections were incubated with primary antibodies diluted in blocking solution at 4 °C overnight (Table 1). Slides were washed six times for 10 min with 1xPBS, incubated with secondary antibodies diluted in blocking solution for 2 h at room temperature, and washed six times with 1xPBS. Nuclei were stained with DAPI (1:10.000 in 1xPBS) for 2 min. After additional washing with 1xPBS for 10 min, slides were mounted with 50 μl Mowiol (Sigma-Aldrich) and stored at 4 °C.

**Table 1:** Primary antibodies

Antigen	Species	Company	Dilution	Catalog number
Calbindin D-28k	Mouse	Swant	1:300	300
Doublecortin	Guinea pig	Millipore	1:1000	ab2253
FLAG	Mouse	Sigma	1:1000	F1804
GFAP	Mouse	Sigma	1:500	G3893
GAPDH	Mouse	Santa Cruz Biotechnologies	1:1000	SC32233
Iba	Goat	Abcam	1:500	ab5076
NeuN	Mouse	–	1:20	–
Nestin	Mouse	Millipore	1:500	MAB353
NeuroD1	Goat	Santa Cruz Biotechnologies	1:500	sc-1084
Parvalbumin	Mouse	Swant	1:500	235
Sox2	Goat	Santa Cruz Biotechnologies	1:500	sc17320
Sox10	Guinea pig	–	1:500	–
TCF4	Rabbit	Abcam	1:500	ab130014

Adult brains were coronally cut at 40 μm thickness using a sliding microtome. Immunofluorescent stainings were performed on free-floating slices. Slices were washed three times with TBS, blocked in TBS containing 0.25% Triton X-100 and 3% normal donkey serum (TBS++), and incubated with primary antibodies in TBS++ for 72 h at 4 °C. Tissue was washed with TBS at room temperature, blocked with TBS++ for 30 min at room temperature, and subsequently incubated with the secondary antibodies diluted in TBS++ overnight at 4 °C or for 2 h at RT. After washing in PBS, nuclei were stained with DAPI and sections were mounted on coverslips with Aqua poly mount (Polysciences).

### Validation of TCF4 antibody

The *TCF4* knockdown construct was generated cloning the *TCF4* forward and reverse oligonucleotides TCF4 forward: 5′-CCGGGCTGAGTGATTTACTGGATTTCTCGAGAAATCCAGTAAATCACTCAGCTTTTT-3′; and *TCF4* reverse: 5′-CGAAAAAGCTGAGTGATTTACTGGATTTCTCGAGAAATCCAGTAAATCACTCAGC-3′.

CCGG into the PWXG1 expression vector (gift from X. Zhao, University of Wisconsin, Madison). The TCF4 expression construct was generated by cloning the human *TCF4* coding sequence fused with a tandem Strep II tag and a FLAG tag on the C-terminus into the CAG-IRES-GFP expression vector [43, 44]. The resulting CAG-TCF4-IRES-GFP was either transfected alone or together with the *TCF4* knockdown construct in HEK 293T cells (ATCC, Wesel, Germany; CRL-3216), and the proteins were extracted 2 days post-transfection. Protein extract obtained from HEK 293T cells transfected with a GFP control construct (CAG-GFP-IRES-GFP) served as a control. Cells were lysed in RIPA buffer (50 mM Tris pH 8, 150 mM NaCl, 1 mM EDTA, 1% NP-40, 0.1%SDS, 0.5% S-DOC,1x protease inhibitor cocktail-EDTAfree-Roche). The extracts were separated in a 10% SDS-PAGE gel. Gels underwent wet transfer onto a nitrocellulose membrane. Membranes were blocked in 5% *w*/*v* skim milk (Sigma Aldrich) in TBS with 0.1% Tween 20 (TBS-T). Incubation with primary antibodies diluted in blocking solution was performed overnight at 4 °C and was followed by washing with TBS-T. Secondary antibodies were diluted in blocking solutions and incubated with the membranes for at least 1 h at room temperature followed by washing with TBS-T. Membranes were treated with Clarity Western Enhanced Chemiluminescence (ECL) Substrate (Bio-Rad) and visualized with ChemiDoc XRS+ System (Bio-Rad). Images were processed using ImageLab 5.2.1 Setup (Bio-Rad).

### Imaging

For embryonic sections, fluorescence signal was detected with an AF6000 Modular Systems Leica fluorescent microscope and documented with a SPOT-CCD camera and the Leica software LAS AF (Version 2.6.0.7266; Leica Microsystems, Wetzlar Germany). For comparison of expression levels, settings were kept constant. For expression and co-localization analysis in the adult murine brain, fluorescence signal was detected using a Zeiss LSM 780 confocal microscope with four lasers (405, 488, 550, and 633 nm) and × 20, × 40, × 63 objective lens. Images were processed using ImageJ.

### Analysis of Tcf4 expression levels

For evaluation of expression levels in the murine brain, images were taken with fixed settings. Expression was categorized into four categories, i.e., high, moderate, low, and not detected, based on intensity of TCF4-immunoreactivity. For comparison of neurodevelopmental TCF4 expression between species, mRNA expression data from selected brain areas of non-human primates and humans were extracted from the NIH Blueprint Non-Human Primate (NHP) Atlas (http://www.blueprintnhpatlas.org) and the BrainSpan Atlas (BrainSpan: Atlas of the Developing Human Brain [Internet]. Funded by ARRA Awards 1RC2MH089921-01, 1RC2MH090047-01, and 1RC2MH089929-01. © 2011. Available from: http://www.brainspan.org/lcm/search?search_type=user_selections), respectively. The NIH Blueprint Non-Human Primate (NHP) Atlas provides microarray-based mRNA expression data of non-human primate neural development from E40–0 months. Microarray data of TCF4 expression in select brain regions was extracted and arranged by ascending age for documentation. The BrainSpan: Atlas of the Developing Human Brain provides microarray based mRNA expression data of human neural development from post conception week 15–21. Microarray data of TCF4 expression in select brain regions was extracted and arranged by ascending age for documentation. For comparison of TCF4 expression between human and mice during adulthood, TCF4 mRNA expression data in the adult human brain was extracted from the Allen Human Brain Atlas [23]. Heatmaps for mRNA expression were manually compared to the immunoreactivity score.

### Cortical thickness measurements

The diameter of the cortex was determined in six areas of the cortex, corresponding to the anatomical divisions documented in the Allen Mouse Brain Atlas [45] Posterior parietal association area (PTLp), Somatosensory area (SSp), rostral and caudal primary somatosensory area-barrel field (SSp-bfd), rostral and caudal primary somatosensory area-trunk (SSp-tr). Measurements were performed using ImageJ (version 2.0.0). Because of the variability of the pial surface, we determined the cortical thickness, as the diameter between layer II/III and the white matter. Five measurements were taken per area in each animal (*n* = 3 for *TCF4ex4*^*WT/WT*^; *n* = 6 for *TCF4ex4*^*LacZ/WT*^) and averaged prior to statistical analysis. Statistical significance was determined using the Mann-Whitney *U* test,*p < 0.05.

### Volume measurement of the dentate gyrus

DG volumes were calculated after measuring the DG area of six consecutive 40-μm thick coronal mouse brain slices spanning a distance of 240 μm from the rostral to caudal hippocampus. Measurements were performed using ImageJ (version 2.0.0) [46], *n* = 3 for TCF4ex4^WT/WT^; *n* = 6 for TCF4ex4^LacZ/WT^. Statistical significance was determined using the Mann-Whitney *U* test,*p < 0.05.

### Patients

cMRIs were obtained from two female individuals with confirmed PTHS. Individual 1 is a 5-year-old girl carrying the de novo mutation c.1880-11A>G in *TCF4* which was confirmed to lead to aberrant splicing and is therefore predicted to result in frameshifting and truncation of the protein (p.(Glu623Valfs*87)). The girl has severe ID with a walking age of 4 years 9 months and lack of speech. She has PTHS typical facial features, behavioral anomalies, sleeping difficulties, constipation, and myopia. Her head circumference is on the 10th centile, and no seizures or breathing anomalies occurred so far. Individual 2 is a 7.5-year-old girl carrying the splice site mutation c.655G>A, predicted to result in frameshifting and truncation of the protein (p.(Asp219Glyfs*37)). She could walk with 2 years 9 months and speaks single words and 2–3-word sentences. She has severe constipation, muscular hypotonia, recurrent infections, and typical facial features. Her head circumference in on the 10th centile, and no seizures or breathing anomalies occurred so far. The individuals had been recruited for a study to unravel the genetic causes for developmental disorders, which was approved by the ethics committee of the Medical Faculty of the Friedrich-Alexander-Universität Erlangen-Nürnberg. All participating families had given their informed consent.
